# Vascularised versus non-vascularised bone graft for scaphoid nonunion: Meta-analysis of randomised controlled trials and comparative studies

**DOI:** 10.1016/j.jpra.2022.12.001

**Published:** 2022-12-17

**Authors:** Yuki Fujihara, Michiro Yamamoto, Satoki Hidaka, Ai Sakai, Hitoshi Hirata

**Affiliations:** a Department of Orthopaedic and Hand Surgery, Nagoya Ekisaikai Hospital; b Department of Hand Surgery, Nagoya University Graduate School of Medicine, Nagoya, Japan

**Keywords:** Bone graft, Scaphoid nonunion, Vascularised, Non-vascularised, VBG, Vascularised bone graft, NVBG, Non-vascularised bone graft

## Abstract

**Background:**

Numerous studies have investigated surgical techniques for vascularised bone graft (VBG) for scaphoid nonunion; however, their efficacies remain unclear. Thus, to estimate the union rate of VBG for scaphoid nonunion, we performed a meta-analysis of randomised controlled trials (RCTs) and comparative studies.

**Methods:**

A systematic search was conducted using PubMed, Scopus, Web of Science, and Cochrane Central Register of Controlled Trials. The search formula was as follows: ((scaphoid nonunion) OR (scaphoid pseudarthrosis)) AND (bone graft). Only RCTs were used in the primary analysis, and comparative studies, including RCTs, in the secondary analysis. The primary outcome was nonunion rate. We compared the outcome between VBG and non-vascularised bone graft (NVBG), pedicled VBG and NVBG, and free VBG and NVBG.

**Results:**

This study included a total of 4 RCTs (263 patients) and 12 observational studies (1411 patients). In the meta-analyses of both RCTs only and RCTs and other comparative studies, no significant difference in nonunion rate was found between VBG and NVBG (summary odds ratio [OR], 0.54; 95% confidence interval [CI], 0.19–1.52 and summary OR, 0.71; 95% CI, 0.45–1.12), respectively. The nonunion rates of pedicled VBG, free VBG, and NVBG were 15.0%, 10.2%, and 17.8%, respectively, and no significant difference was found.

**Conclusions:**

Our results indicated that the postoperative union rate in NVBG is similar to that in VBG; thus, NVBG could be the first choice of treatment for scaphoid nonunion.

## Introduction

Scaphoid fractures are sometimes overlooked because of their relatively subtle symptoms and peculiar shape, which in turn result in nonunion.^1^^,^^2^ Untreated scaphoid nonunion could progress to arthritic change, which is referred to as scaphoid nonunion advanced collapse wrist. Düppe et al. reviewed the 30-year follow-up results of scaphoid fractures treated with thumb spica short arm casts and found that 60% of the patients with nonunion showed radiocarpal osteoarthrosis and only 2% of those without nonunion demonstrated degenerative changes.^3^ Thus, a scaphoid nonunion should be treated surgically with internal fixation accompanied with bone graft to achieve bony union.^1^^,^^2^^,^^4^^,^^5^

However, the preferred donor site for bone grafting remains to be clearly established. ^2^^,^^4^^,^^6^^,^^7^ For scaphoid nonunion treatment, there are three types of bone grafting techniques: conventional grafting, pedicled vascularised bone grafting (VBG), and free VBG. The VBG technique was initially considered an ideal technique and was expected to contribute to a 100% union rate; however, its union rate did not meet expectations. Chang et al., Hirche et al., and Straw et al. reported that the union rates of VBGs for scaphoid nonunion were at 75%, 50%, and 12%, respectively. 8, 9, 10 Merrell et al. conducted a systematic review and meta-analysis and showed that the outcome of VBG is preferable to that of wedge grafting.^11^ However, they included not only comparative studies but also case series, and the analysis was a simple summation of the number of patients with bony union after VBG. Thus, the quality of the evidence was limited. Moreover, the latest findings in the subject area are not considered as the paper was published in 2002. Currently, the utility of VBG remains unknown. Hence, we hypothesised that VBG for scaphoid nonunion showed a superior union rate to non-vascularised bone grafts (NVBGs) and conducted a systematic review and meta-analysis to compare the union rate between VBG and NVBGs in patients with scaphoid nonunion.

## Materials and methods

### Search strategy

Because we used the data extracted from officially published articles, the ethical committee in our hospital waived the need to obtain approval for this study. To manage its heterogeneity, we performed a manual systematic literature search for randomised controlled trials (RCTs) and comparative studies following a predefined protocol and in accordance with the Preferred Reporting Items for Systematic Reviews and Meta-Analyses checklist.^12^ Two authors independently reviewed the studies published between January 1, 1984, and September 31, 2020; the studies were from PubMed, Scopus, Web of Science, and Cochrane Central Register of Controlled Trials. The search terms were as follows: ((scaphoid nonunion) OR (scaphoid pseudarthrosis)) AND (bone graft). Moreover, we conducted an electronic search of the databases on October 1, 2020. Table 1 presents the inclusion and exclusion criteria for the meta-analysis.

**Table 1:** Inclusion and exclusion criteria for article selection.

Inclusion criteria
• A full-length article with sufficient outcome data (i.e., sample size of each group for each result and/or ORs and SE, SD, or 95% CI) (minimum sample size requirement was 10) that can be used in the comparison between patients with scaphoid fracture nonunion treated with VBG and those treated with NVBG. • Studies including at least one patient who underwent VBG and one patient who had NVBG • Sample size >10. • Published between January 1, 1984, and September 30, 2020
Exclusion criteria
• If two studies based on the same topic were published by the same faculty, the older study was excluded. • Review articles, conference papers, or short letters • Studies focusing on patients in a specific category (adolescents, elderly adults, etc.) • More recent study dealing with the same patient group was published from the same facility

VBG, vascularised bone graft; NVBG, non-vascularised bone graft; OR, odds ratio; SE, standard error; SD, standard deviation; CI, confidence interval.

### Data extraction

Two investigators independently extracted the data from eligible studies using predetermined selection criteria. We planned to resolve discrepancies through a discussion with a third investigator; however, this was ultimately not necessary as there were no discrepancies. The studies were systematically assessed for quality and risk of bias by two independent researchers using the risk of a bias assessment tool for non-randomised controlled studies.^13^

The study design data and patient characteristics, including the location of the fracture on the scaphoid, osteosynthesis techniques, mean follow-up period, mean age, and sex, were obtained from the selected articles. Furthermore, we identified the total number of bone graft techniques performed in the operation for scaphoid nonunion and the postoperative nonunion rate. We included the studies with a single bone graft technique for the treatment of nonunion. For multiple studies from the same facility with the same outcomes, we included only the most recent studies.

### Statistical analysis

We calculated the odds ratios (ORs) for binary variables. Conventionally, a higher OR in the VBG cohort indicates a higher nonunion rate. For the primary outcome, we compared the nonunion rate between patients who received VBGs and those who received NVBGs using the data extracted from RCTs only. Additionally, we conducted the same analysis with the data extracted from not only RCTs but also other comparative studies. In the subgroup analysis, we compared the nonunion rate between pedicled VBG and NVBG and between free VBG and NVBG. To minimise the heterogeneity of outcome evaluation, we performed additional subgroup analysis with the studies that adopt outcome evaluation using computed tomography (CT) for all patients or patients suspected to be nonunion. We evaluated the results for heterogeneity using forest plot and I^2^ statistical tests and by comparing the summary ORs using random-effects models. We used funnel plots to evaluate publication bias. Regarding the sample size, we calculated the standard mean difference (Cohen's d).^14^ All statistical analyses were performed with EZR version 1.54 (Saitama Medical Center, Jichi Medical University, Saitama, Japan), which is a graphical user interface for R (The R Foundation for Statistical Computing, Vienna, Austria).^15^ EZR is a modified version of R commander that was designed to add statistical functions frequently used in biostatistics.

## Results

### Study selection

**Figure 1:**
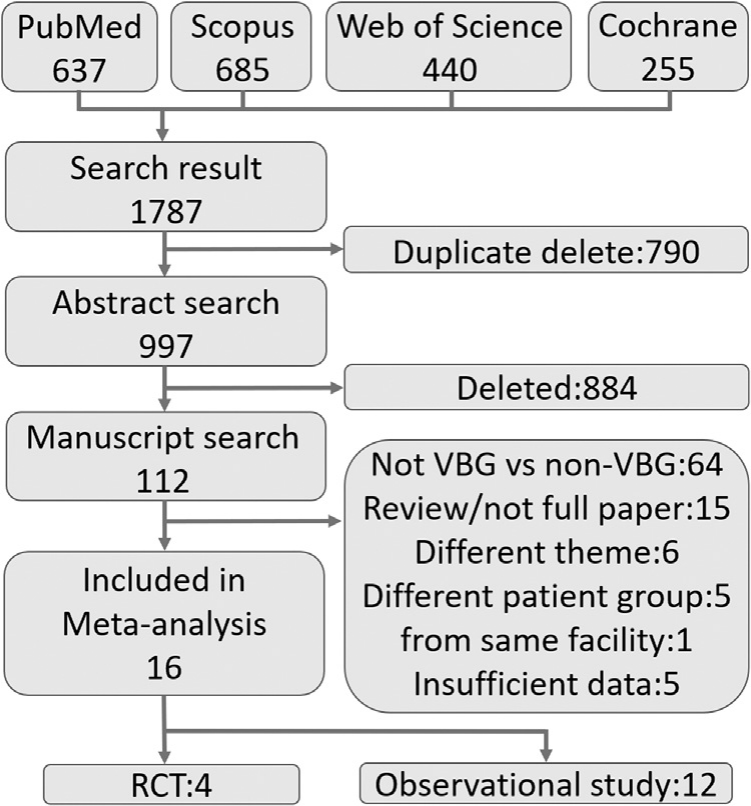
Flowchart of patient selection. We identified 997 studies in the electronic literature search. Based on the inclusion and exclusion criteria, we selected four randomised controlled trials and 12 observational studies for the meta-analysis.

Our search identified 1787 articles, from which 790 duplicate articles were excluded. Based on the inclusion and exclusion criteria, we performed an abstract search and a manuscript search. We identified 16 comparative studies, including 4 RCTs^16^, ^17^, ^18^, ^19^ and 12 observational studies (Figure 1).^20^, ^21^, ^22^, ^23^, ^24^, ^25^, ^26^, ^27^, ^28^, ^29^, ^30^, ^31^ No arthroscopic-assisted bone grafts were included in this study.

### Study characteristics

The characteristics of the patients in the articles included in the meta-analyses are shown in Table 2. Briefly, 89.0% of the patients were males (mean age, 26 years). Nonunion in the scaphoid waist accounted for 57% of the nonunion sites and that in the proximal pole for 36%.

**Table 2:** Detailed characteristics of the patients in the articles included in the meta-analyses.

Author	Year	Study design	Location of fracture	Osteosynthesis	Mean follow-up period	Mean age	Sex (male/female)
Aibinder et al.	2019	Retrospective study	Waist: 80, proximal pole: 38	CS: 69, K-wire: 27, both: 13	16.5 months	25	93/16
Ammori et al.	2019	Retrospective study	Waist: 316, proximal pole: 119, distal pole: 27	None: 4, K-wire: 35, CS: 422, other screw: 1	3–6 months: 179, 6–12 months: 149, 1–2 years: 91, 2 years: 43	26	427/33
Braga-Silva et al.	2008	RCT	Waist: 56, proximal pole: 24	NVBG group HS: 45, VBG group wire: 30, HS: 5	2.8 years	26	56/24
Caporrino et al.	2014	RCT	Waist: 53, proximal pole: 10, others: 12	All K-wire	29.0 months	28	71/4
Ciprian et al.	2004	Retrospective study	Waist: 4, proximal pole: 17	Osteosynthesis pins (n=2)	No description	31	18/3
Fox et al.	2015	Retrospective study	Proximal pole: 18	No description	14 CT cases: 96 days, 16 X-ray cases: 149 days	18	16/2
Garcia et al.	2014	Retrospective study	Waist: 12, proximal pole: 5, distal pole: 2	Trimed: 11, combined Acutrak Mini and Acutrak 2 Micro: 8	3.6 months for bony union	21	18/1
Guzzini et al.	2019	Retrospective study	Proximal pole necrosis	No description	12.5 months	30	23/9
Jaminet et al.	2019	Retrospective study	Proximal 3rd: 126, middle 3rd: 130, distal 3rd: 30	Mini-HS and/or K-wire	No description	26	258/28
Kömürcü et al.	2001	Retrospective study	Proximal: 6, middle: 20, distal: 16	K-wire: 15, AO CS: 8, HS: 19	2.4 years	24	35/7
Küntscher et al.	2001	Retrospective study	Proximal pole	K-wire and HS	14.5 months	24	24/1
Pechlaner et al.	1990	Retrospective study	No description	K-wire (for VBG): 35, HS (for NVBG): 18	>2 years	27	No description
Raju et al.	2011	RCT	Proximal pole: 13, waist: 12, distal pole: 8 (including HS group)	K-wire	28 months	28	27/6 (including HS group)
Ribak et al.	2010	RCT	Proximal pole: 37, middle: 47, distal pole: 2	K-wire	23.1 months	No description	No description
Schaller et al.	1993	Retrospective study	No description	HS vs. Matti-Russe vs. VBG	No description	No description	No description
Smeraglia et al.	2020	Prospective study	No description	K-wire (for VBG): 9, HS (for NVBG): 12	No description	27	All male

HS, Herbert screw; CS, cannulated screw; VBG, vascularised bone graft; NVBG, non-vascularised bone graft; 1,2 ICSRA, 1,2 intercompartmental supraretinacular artery; MFC, medial femoral condyle.

### Meta-analysis outcomes

**Figure 2:**
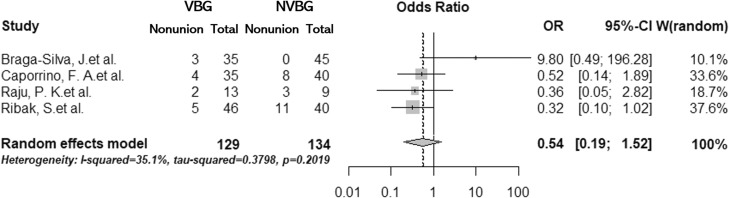
Forest plot for the meta-analysis of randomised controlled trials. Non-vascularised bone graft was similar to vascularised bone graft. VBG, vascularised bone graft; NVBG, non-vascularised bone graft.

**Figure 3:**
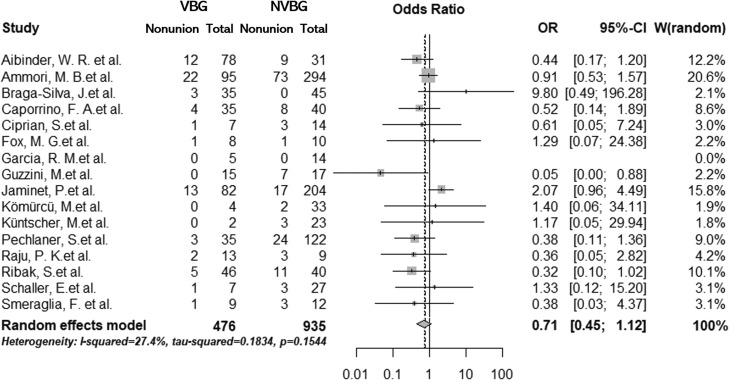
Forest plot for the meta-analysis of comparative studies. Results of the meta-analysis of comparative studies were consistent with those of the meta-analysis of randomised controlled trials. VBG, vascularised bone graft; NVBG, non-vascularised bone graft.

**Figure 4:**
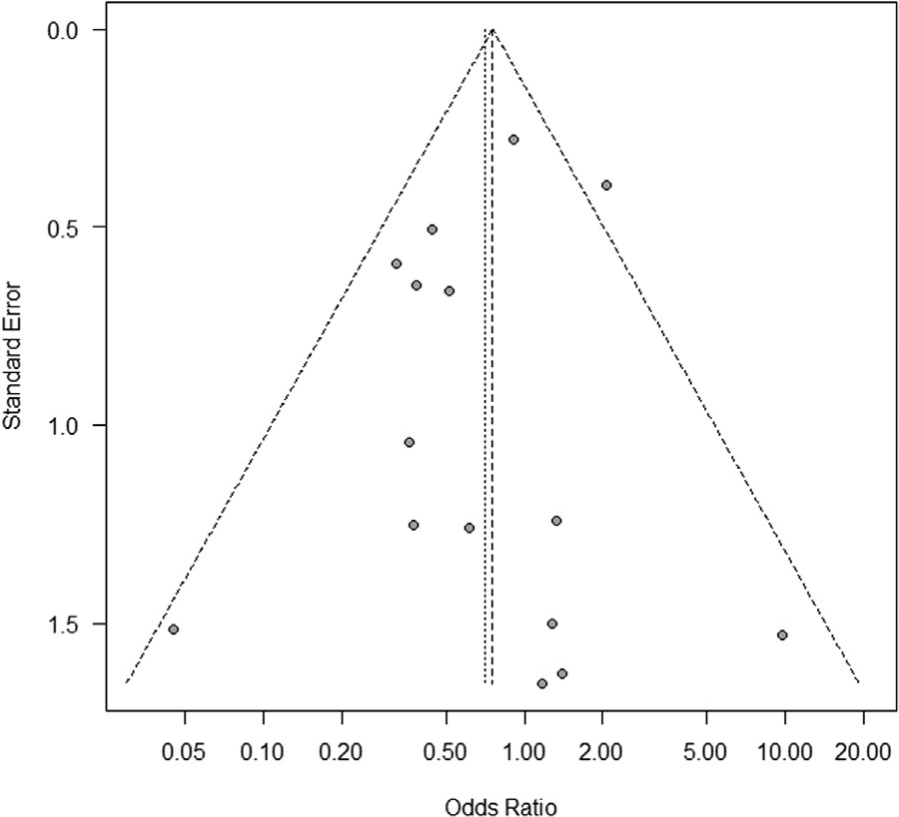
Funnel plot for the meta-analysis with randomised controlled trials. Significant publication bias was not found (p = 0.45).

**Figure 5:**
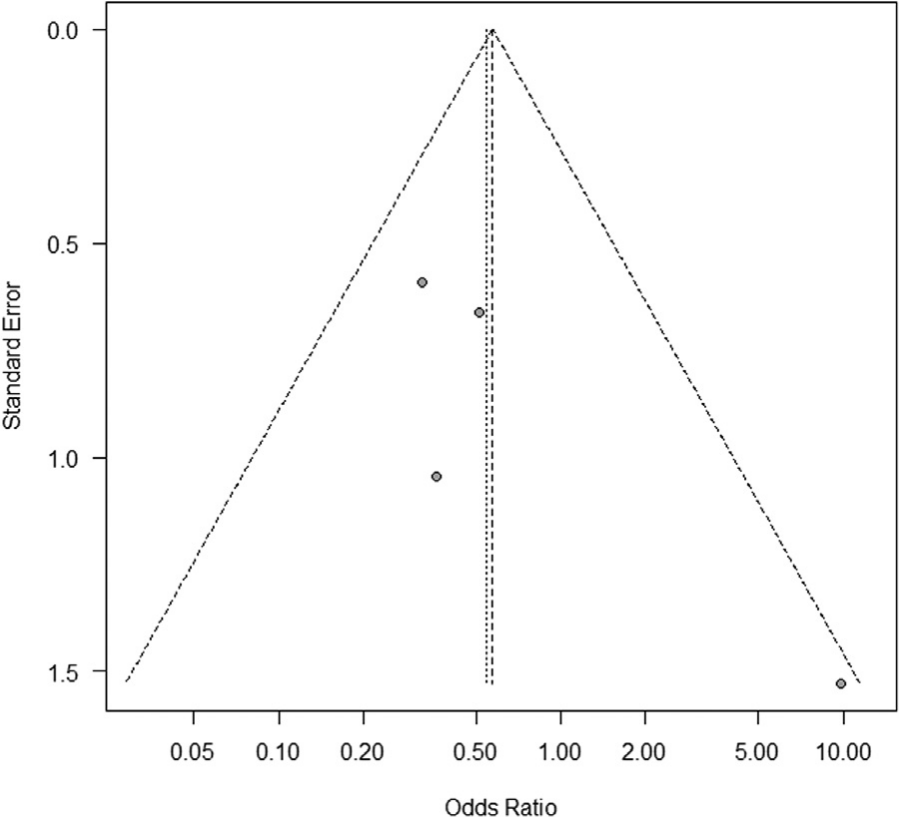
Funnel plot for the meta-analysis of all comparative studies. No significant publication bias was observed (p = 0.51).

In the primary analysis, which included RCTs only, we found no significant difference in nonunion rate between all VBGs and NVBG (summary OR, 0.54; 95% confidence interval [CI], 0.19–1.52) (Figure 2). Similar results were found in the secondary analysis, which included all comparative studies (summary OR, 0.71; 95% CI, 0.45–1.12) (Figure 3). These two analyses showed relatively low heterogeneities (I^2^ = 35% and 27%, respectively). No significant publication bias was detected in the two analyses (primary, p = 0.50; secondary, p = 0.45) (Figures 4 and 5). The outcomes of patients in each article are shown in Table 3. A total of 399 pedicled VBG, 68 free VBG, and 923 NVBG were performed, and the nonunion rates were 15%, 10%, and 18%, respectively. Pedicled VBGs were harvested from either the volar or dorsal radius, and most of the free VBGs were harvested from the medial femoral condyle. Neither pedicled VBG nor free VBG was superior to NVBG regarding nonunion rate (summary OR, 0.82; 95% CI, 0.56–1.20; and summary OR, 0.37; 95% CI, 0.07–1.87, respectively). In all selected articles, there were two studies (Jaminet, P. et al. 2019 and Smeraglia, F. et al.)^26^^,^^31^ that adopted CT outcome evaluation for all patients and three studies (Aibinder et al. 2019, Caporrino et al. 2014, and Küntscher et al. 2001)^17^^,^^20^^,^^28^ that adopted repeated CT outcome evaluation for patients who were suspected of nonunion. We performed subgroup analysis with these five articles, which resulted in similar results to the other outcomes (summary OR, 0.80; 95% CI, 0.55–1.59). Calculated Cohen's d for the meta-analysis of all studies, RCTs, and studies with CT outcome evaluation were 1, 0.83, and 1.00, respectively, which proved to be a sufficient sample size.

**Table 3:** Techniques and outcomes of bone graft.

Author	Year	Pedicled VBG total number	Pedicled VBG total nonunion	Type of pedicled VBG	Free VBG total number	Free VBG nonunion	Type of free VBG	NVBG total number	NVBG nonunion	Type of NVBG
Aibinder et al.	2019	33	7	1,2-ICSRA	45	5	MFC: 45	31	9	Iliac crest bone graft
Ammori et al.	2019	89	20	Vascularized local bone flap	6	2	Free VBG: 6	294	73	Distal radius/ulna bone graft: 122, iliac crest: 172
Braga-Silva et al.	2008	35	3	1,2-ICSRA	NA	NA	NA	45	0	Iliac crest
Caporrino et al.	2014	35	4	1,2-ICSRA	NA	NA	NA	40	8	Distal radius NVBG
Ciprian et al.	2004	7	1	From radius	NA	NA	NA	14	3	Iliac crest: 10, radius: 3, ulna: 1
Fox et al.	2015	8	1	1,2-ICSRA	NA	NA	NA	10	1	Autogenous bone graft
Garcia et al.	2014	3	0	Capsular-based vascularized distal radius graft	2	0	MFC: 2	14	0	Corticocancellous autograft from iliac crest
Guzzini et al.	2019	NA	NA	NA	15	0	MFC: 15	17	7	Bone grafts
Jaminet et al.	2019	82	13	Palmar vascularized bone graft	NA	NA	NA	204	17	Iliac crest bone graft
Kömürcü et al.	2001	4	0	Pronator quadratus pedicled Bone graft	NA	NA	NA	33	2	Tricortical iliac crest
Küntscher et al.	2001	2	0	Radius	NA	NA	NA	23	3	Cancellous bone graft: 19, interpositional iliac bone graft: 4
Pechlaner et al.	1990	35	3	Pechlaner-Hussl vascular Pedicle bone: transplant	NA	NA	NA	122	24	Matti-Russe I: 86, Russe II: 18, HS fixation: 18
Raju et al.	2011	13	2	Kuhlmann's VBG	NA	NA	NA	9	3	Matti-Russe
Ribak et al.	2010	46	5	VBG from dorsal radius	NA	NA	NA	40	11	Distal radius
Schaller et al.	1993	7	1	VBG from dorsal radius	NA	NA	NA	27	3	Matti-Russe
Smeraglia et al.	2020	9	1	Kuhlmann's VBG	NA	NA	NA	12	3	Iliac bone graft
Total		399	60		68	7		923	164	

HS, Herbert screw; CS, cannulated screw; VBG, vascularized bone graft; 1,2 ICSRA, 1,2 intercompartmental supraretinacular artery; MFC, medial femoral condyle; NA, not applicable.

### Risk of bias

The risk of bias is summarised in Table 4. Regarding participant selection, two studies included patients from different study periods,^30^^,^^31^ and one study selected patients from a computer database.^22^ None of the studies controlled for confounding bias, and no performance bias was found. One study had a blinded outcome evaluation,^24^ and a musculoskeletal radiologist was involved in the outcome evaluation of one study.^26^ Six studies evaluated bony union with objective measurements using CT or magnetic resonance imaging. Outcome evaluation was performed using plain radiographs in four studies, and two studies did not mention their outcome evaluation strategy. Two studies excluded more than 20% of the study subjects because of incomplete outcome data,^21^^,^^22^ and one study did not mention incomplete outcome data.^29^ Although no studies referenced a published protocol with predefined outcomes, the expected outcomes were presented in all 12 observational studies.

**Table 4:** Risk of bias in the included non-randomized controlled studies (RoBANS).

Author	Year	Journal	Study design	Selection of participants	Confounding variables	Measurement of exposure	Blinding of outcome assessments	Incomplete outcome data	Selective outcome reporting
Aibinder et al.	2019	Hand (N Y)	Retrospective study	Low	High	Low	Low	Low	Low
Ammori et al.	2019	J Hand Surg Eur Vol	Retrospective study	Low	High	Low	High	High	Low
Ciprian et al.	2004	J Radiol	Retrospective study	High	High	Low	Low	High	Low
Fox et al.	2015	Skeletal Radiol	Retrospective study	Low	High	Low	High	Low	Low
Garcia et al.	2014	J Hand Surg Am	Retrospective study	Low	High	Low	Low	Low	Low
Guzzini et al.	2019	Acta Biomed	Retrospective study	Low	High	Low	?	Low	Low
Jaminet et al.	2019	Eplasty	Retrospective study	Low	High	Low	Low	Low	Low
Kömürcü et al.	2001	J South Orthop Assoc	Retrospective study	Low	High	Low	High	Low	Low
Küntscher et al.	2001	Unfallchirurg	Retrospective study	Low	High	Low	Low	Low	Low
Pechlaner et al.	1990	Unfallchirurg	Retrospective study	Low	High	Low	High	?	Low
Schaller et al.	1993	Handchir Mikrochir Plast Chir	Retrospective study	High	High	Low	?	Low	Low
Smeraglia et al.	2020	J Biol Regul Homeost Agents	Prospective study	High	High	Low	Low	Low	Low

?, unclear risk of bias.

## Discussion

In this meta-analysis, a comparison of the union rates between VBG and NVBG was performed. In the analyses of both comparative studies and RCTs alone, VBG was not superior to NVBG. Free VBG showed the lowest nonunion rate among the bone graft procedures; however, the difference was not statistically significant.

VBG for scaphoid nonunion was first performed by Roy-Camille in 1965; he applied a pedicled VBG from the palmar tubercle of the scaphoid to its nonunion site with an abductor pollicis brevis muscle pedicle.^32^ Currently, various VBG procedures using grafts harvested from the volar or dorsal aspect of the radius, second metacarpal base, medial femoral condyle, or iliac crest have been developed.^33^, ^34^, ^35^, ^36^, ^37^, ^38^, ^39^ They believed the efficacy of VBG for scaphoid nonunion, because of its peculiar vascularity pattern, first described by Gelberman in 1980, frequently caused avascular nonunion.^40^

However, recent studies have reported contradictory outcomes.^41^^,^^42^ Rancy et al. have reported a case series of patients with scaphoid nonunion who underwent NVBG with Herbert screw fixation.^41^ They evaluated the vascularity of the proximal pole with preoperative magnetic resonance imaging, assessed intraoperative bleeding points, and performed histopathological analysis of the cancellous bone and concluded that proximal pole infarction is decidedly rare and that VBG is seldom required. Moreover, they reviewed the literature and summarised the systematic reviews, case series, and RCTs regarding VBG for scaphoid nonunion and cast doubt on the efficacy of VBG. ^4232^ Their conclusion was supported by our results, which included integrated data. In our study, we conducted meta-analyses of two different groups of studies, i.e., RCTs only and all comparative studies. As there were only four RCTs included, an additional meta-analysis including all comparative studies for the same topic was performed. Although several meta-analyses included four or fewer articles, 43, 44, 45, 46 we believe that the number of articles included in a meta-analysis is essential.

The results of the subgroup analyses indicated that both pedicled VBG and free VBG were not significantly superior to NVBG. Moreover, free VBG showed the lowest nonunion rate, and a few cases were treated with free VBG, which may explain the absence of a statistically significant difference. Further accumulation of cases may result in different conclusions.

Although this study was conducted using a systematic protocol, a few limitations still existed. First, this study did not assess any patient-reported outcomes, functional outcomes, the duration from bone grafting to union, or radiographic parameters. Each study applied various types of measurement tools for these outcomes; thus, the analysis would have been difficult. In this study, we focused on the biological aspects of bone grafts for scaphoid nonunion. Second, we consolidated various types of VBGs. Data consolidation is a typical limitation of a meta-analysis as some of the specific data from the original sources may be lost. Nevertheless, a previous meta-analysis compared the efficacy of nine VBGs and concluded that no significant difference was found among the VBGs.^47^ Third, we did not assess the type of osteosynthesis, which is automatically determined based on the fragment size or the used bone graft; thus, osteosynthesis cannot be an independent variable. Although several studies have reported that no specific fixation methods can contribute to an increased union rate, ^48^^,^^49^ the lack of such an assessment may reduce the quality of this study. Fourth, the treatment methods varied depending on the type of fracture, especially in the non-randomised comparative studies. Fifth, we could not determine the uniformity of the part of the fracture because most of the selected studies did not report the detailed relationship between the nonunion rate, part of the fracture, and used techniques. Sixth, we did not evaluate the preoperative vascularity of the proximal fragment. Although this bias was managed by undertaking a meta-analysis of RCTs exclusively, it exists in the meta-analysis of all comparative studies and thus may influence the overall outcome.

## Conclusion

Despite the limitations, we performed a thorough review of the literature and provided further information on the role of VBG in scaphoid nonunion treatment. Free VBG showed the lowest nonunion rate, and current evidence showed that VBG is not significantly superior to NVBG. Hence, the results of this study suggest that the efficacy of VBG should not be overestimated and that patients may benefit from treatment procedures that are not excessively invasive.

## Conflict of interest statement

There are no conflicts of interest in the article.

## Appendix Supplementary materials

Image, application 1
